# Severely impaired microvascular reactivity in diabetic patients with an acute coronary syndrome

**DOI:** 10.1186/s12933-016-0385-6

**Published:** 2016-04-20

**Authors:** Nikolaos Östlund Papadogeorgos, Gun Jörneskog, Mattias Bengtsson, Thomas Kahan, Majid Kalani

**Affiliations:** Department of Clinical Sciences, Karolinska Institutet, Danderyd Hospital, Stockholm, Sweden; Dept of Cardiology, Danderyd University Hospital Corp, 182 88 Stockholm, Sweden

**Keywords:** Microvascular reactivity, Coronary artery disease, Acute coronary syndrome, Diabetes mellitus, Laser Doppler fluxmetry

## Abstract

**Background:**

Microvascular function is impaired in patients with stable coronary artery disease. The aim was to study microvascular function in patients with diabetes and acute coronary syndrome (ACS).

**Methods:**

Microvascular function was evaluated in 83 patients by laser Doppler fluxmetry (LDF) [PU; perfusion unit, median (interquartile range)] measuring resting LDF and peak LDF following a six min heating of the skin to 44 °C at the foot, respectively. All patients with ACS and without previously known diabetes underwent oral glucose tolerance test. Thirty-nine patients with type 2 diabetes mellitus free from coronary artery disease served as controls.

**Results:**

Peak LDF was significantly (P = 0.03) lower in patients with ACS and diabetes (n = 22; 72 (52)) and diabetes without coronary artery disease (n = 39; 69 (51)) as compared to patients with ACS without diabetes (n = 46; 97 (60)), and patients without ACS (n = 15; 140 (121)), respectively. Patients with ACS (n = 68) had significantly (P = 0.04) lower peak LDF (92 (49)) as compared to patients without ACS (n = 15) (140 (121)).

**Conclusion:**

Microvascular reactivity is severely impaired in patients with diabetes and ACS. Diabetes has a major influence on microvascular function in patients with coronary artery disease.

## Background

There is compelling evidence for a link between abnormalities of the microvascular function and the pathogenesis of cardiovascular disease [1–4]. Furthermore, microcirculation is essential for adequate blood supply and oxygenation to tissues, in particular when exposed to ischaemia. Optimal microvascular reactivity in response to ischaemia is crucial to limit the extent of tissue injury. Strong associations between endothelial dysfunction in the peripheral circulation and both atherosclerotic risk and future cardiovascular events have been described. Most of these studies have investigated vascular function in the brachial artery, providing large vessel function [5–8]. There are a few studies of peripheral blood flow, e.g., skeletal muscle of the calf and subcutaneous blood flow, in the acute phase of myocardial infarction [9, 10]. More recently, a few studies of skin microcirculation in patients with coronary artery disease have been published, showing disturbed skin microcirculation in these patients [11–14]. Furthermore, an impaired microvascular endothelial function has been shown in patients with early-onset coronary artery disease [15, 16]. A link between skin microcirculation and myocardial microvascular function has been described indicating that peripheral microcirculatory variables may be useful for non-invasive assessment of myocardial ischaemia [17]. A potential link between microvascular function in the skin and in the myocardium facilitates studying the pathophysiology of myocardial microvascular dysfunction during myocardial ischaemia. Impaired insulin resistance has been associated with coronary microcirculatory dysfunction in patients with ST segment elevation myocardial infarction [18]*.*

However, the influence of impaired glucose tolerance and/or diabetes on skin microcirculation has not been studied in patients with acute coronary syndrome (ACS). Impaired glucose tolerance or type 2 diabetes is present in as many as two-thirds of all patients with an ACS and is associated with poor clinical outcome [19, 20].

The aim of the present study was to investigate skin microvascular reactivity in patients with ACS, and to evaluate the influence of impaired glucose tolerance and/or diabetes on skin microvascular reactivity.

## Methods

### Patient population

Eighty-three consecutive patients admitted to our institution with suspected coronary artery disease during a period of 2 months were investigated. Included were patients with verified or suspected ACS in need of observation in the cardiology department. Patients with unstable haemodynamic parameters, e.g., cardiogenic shock and uncompensated congestive heart failure, and history of peripheral arterial occlusive disease were excluded. Thirty-nine patients with type 2 diabetes mellitus free from coronary artery disease previously investigate in diabetes mellitus and diastolic dysfunction study served as controls [21]. An ACS was defined according to the European Society of Cardiology definition [22]. Diabetes mellitus was defined according to the World Health Organization definition, and the fasting plasma glucose (FBG) criteria according to the American Diabetes Association [23]. The study protocol, conducted according to the declaration of Helsinki, was approved by the local human ethics committee of Karolinska Institutet. Oral and written informed consent was obtained from all patients.

### Microcirculation and biochemical analysis

The vascular investigations were performed within 3 days of admission but not during the first 24 h. All investigations were performed in the morning, in the supine position after 30 min of acclimatization. Patients were asked to refrain from coffee and smoking for at least 8 h before the investigation. Brachial systolic and diastolic blood pressures were measured by the Riva Rocci method. Ankle systolic blood pressure was measured with a pen Doppler device over the dorsal pedal and posterior tibial arteries. Arm and ankle blood pressures were measured three times and averaged and an ankle/brachial-index was calculated.

All studies of skin microvascular reactivity were conducted in a temperature-controlled room (22 ± 1.0 °C). Following measurement of skin temperature, skin microcirculation was evaluated by laser Doppler Fluxmetry (LDF) (PeriFlux 4001 Master, Perimed, Järfälla, Sweden) at the dorsum of the foot. Following 3 min measurement of resting LDF, peak skin microvascular vasodilatation was assessed following 6 min of heating of the skin under the LDF probe to 44 °C (peak LDF) (PeriTemp 4005 with thermostatic probe PF 457, Perimed, Järfälla, Sweden). Day-to-day coefficient of variation for peak LDF during local heating of skin to 44 °C at the foot level has been investigated in 5 healthy subjects at our laboratory and was 18 %.

Patients diagnosed with ACS and without previously known diabetes underwent a standardised 75-g oral glucose tolerance test (OGTT) according to WHO criteria prior to discharge, not earlier than on day 4 following admission. Patients were classified as having diabetes if the 2-h post load plasma glucose concentration exceeded 11.0 mmol/l. Impaired glucose tolerance (IGT) was defined as a 2-h post load blood glucose between 8.7 and 11.0 mmol/l.

Fasting plasma glucose, glycated haemoglobin (HbA1c), total serum cholesterol, low density lipoprotein (LDL), high density lipoprotein (HDL) concentrations and triglycerides (TG) were assessed at day 3 post admission by standard methods, according to local laboratory routines. HbA1c was analysed by the Mono S method only in patients with diabetes.

### Statistical analysis

Data are presented as mean values ±SD if not otherwise stated. Microcirculation data are expressed as medians and interquartile range. Group comparisons were made by the Mann–Whitney rank sum test and the Kruskal–Wallis test. Associations were assessed by the Spearman rank correlation. A two-sided probability (P) value <0.05 was considered significant.

## Results

The basal characteristics of the 83 admitted patients, divided according to the presence of ACS and diabetes, and 39 patients with type 2 diabetes free from coronary artery disease are shown in Table 1. An ACS was confirmed in 68 admitted patients. Of these, 21 patients had a known type 2 diabetes, and of the remaining 47 patients who underwent OGTT, one patient was diagnosed with type 2 diabetes and ten with IGT. None of the patients had type 1 diabetes. The 15 patients without ACS had all normal fasting plasma glucose levels. These patients had no evidence of significant coronary artery disease. All patients were in NYHA class I. There was a trend for higher age and serum creatinine levels in patients with ACS, as compared to those without ACS. Patients with ACS and diabetes had higher body mass index, as compared to other groups, while levels of total cholesterol and LDL were lower in patients with ACS and diabetes. Statin therapy was more common in patients with diabetes (data not shown).

**Table 1:** Baseline characteristics of 83 patients divided into three groups according to presence of acute coronary syndrome (ACS) and diabetes (DM), and 39 patients with diabetes mellitus free from coronary artery disease (CAD)

	ACS+/DM+	ACS+/DM−	ACS−/DM−	ACS−/DM+	P
n	22	46	15	39	
Age (years)	71 ± 10	72 ± 11	65 ± 12	61 ± 18^a, b^	0.03
Male/female	11/11	32/14	9/6	16/23	
Diabetes duration (years)	12 ± 9	–	–	6 ± 4	0.05
Fasting plasma glucose (mmol/L)	7.8 ± 1.2	5.4 ± 0.8^a^	5.2 ± 0.7^a^	7.6 ± 1.4^b, c^	0.02
HbA1c (%)	6.8 ± 1.3	–	–	5.9 ± 1.6^a^	0.04
Smoker/ex-smoker	0/10	8/25	3/8	3/9	
S-creatinine (µmol/L)	110 ± 44	98 ± 33	85 ±± 17	90 ± 30	0.07
Brachial systolic blood pressure (mm Hg)	118 ± 24	115 ± 22	113 ± 24	143 ± 15^a, b, c^	0.02
Ankle blood pressure (mm Hg)	128 ± 37	117 ± 38	142 ± 28	157 ± 20^a, b^	0.03
Ankle brachial index	1.1 ± 0.4	1.1 ± 0.5	1.3 ± 0.4	1.1 ± 0.4	0.06
Body mass index (kg/m^2^)	31 ± 3	25 ± 3^a^	28 ± 3	27 ± 7^a^	0.01
Total cholesterol (mmol/L)	3.9 ± 0.8	4.5 ± 1.3	5.0 ± 1.0^a^	4.7 ± 1.0^a^	0.02
LDL (mmol/L)	2.0 ± 0.6	2.6 ± 1.2	3.2 ± 1.0^a^	2.7 ± 0.9	0.01
HDL (mmol/L)	1.0 ± 0.2	1.2 ± 0.4	1.3 ± 0.3^a^	1.0 ± 0.3^c^	0.01
Triglycerides (mmol/L)	2.1 ± 1.2	1.3 ± 0.7^a^	1.3 ± 0.5^a^	1.4 ± 0.8^a^	0.01

Data are presented as mean values ±SD or number of patients

*HbA1c* glycated haemoglobin, *LDL* low density lipoprotein, *HDL* high density lipoprotein, *TG* triglycerides

vs ACS+/DM+, ^a^P < 0.05; vs ACS+/DM−, ^b^ P < 0.05; vs ACS−/DM−, ^c^ P < 0.05

All patients had stable haemodynamic measures, without any signs of cardiogenic shock or decompensated heart failure at the time of admission and/or vascular investigations.

Ankle blood pressure was lower in patients with ACS, as compared to patients without ACS, while there were no significant differences in ankle/brachial-index between the groups.

**Fig. 1:**
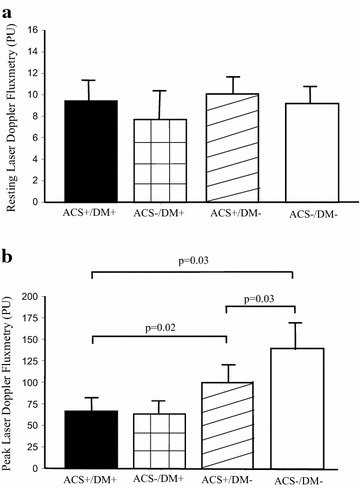
Resting laser Doppler fluxmetry (**a**) and post reactive hyperaemia (peak LDF) (**b**) in patients with ACS and diabetes (ACS+/DM+; n = 22), ACS without diabetes (ACS+/DM−; n = 46), no ACS and no diabetes (ACS−/DM−; n = 15), and diabetes without coronary artery disease (ACS−/DM+; n = 39). Data are depicted as median and quartiles. *PU* perfusion unit

The results of the investigations of skin microvascular reactivity are presented in Fig. 1. There were no significant differences in resting LDF between the groups (Fig. 1a). Significantly lower peak LDF was measured in patients with ACS and diabetes and patients with diabetes free from CAD, respectively, compared to patients with ACS without diabetes and patients without ACS (Fig. 1b). The highest peak LDF was found in the patients without ACS and no diabetes (Fig. 1b). In the ten patients with ACS and IGT, peak LDF was 101 (75) PU, as compared to 72 (52) PU in 22 patients with diabetes (P = 0.07). There were no differences in skin temperature between the groups (data not shown). A weak negative correlation (r = −0.22; P = 0.05) was found between body mass index and peak LDF. Age, blood lipids, fasting plasma glucose, HbA1c, brachial or ankle systolic blood pressure did not relate to peak LDF.

## Discussion

We found that microvascular reactivity is impaired in patients with ACS, and that the impairment is more pronounced in patients with ACS and concomitant type 2 diabetes. Interestingly, data from patients with diabetes free from CAD showed same degree of microvascular dysfunction as in patients with ACS and diabetes. These findings indicate that diabetes plays a major role in the pathogenesis of microvascular dysfunction, irrespective of presence of CAD.

It has been demonstrated in previous studies that functional disturbances in the microvasculature of patients with diabetes is characterised by reduced vasodilatory capacity and impaired nutritive capillary circulation [24, 25]. Vascular endothelial dysfunction, which is closely linked to diabetes and impaired insulin sensitivity, may be a contributing factor to impaired microvascular reactivity. Disturbed microvascular function is important for the diabetes specific complications including diabetic cardiomyopathy [26]. Impaired microvascular reactivity may cause disturbed myocardial perfusion in patients undergoing percutaneous coronary intervention (PCI) for myocardial infarction [27, 28], which may contribute to poor prognosis in these patients [20].

In the present study, the impairment of microvascular reactivity was more pronounced in patients with diabetes, with or without ACS, as compared to patients without diabetes. In addition to acute endothelial dysfunction due to an ACS, hyperglycaemia per se may promote platelet activation, augment plugging of blood cells in the capillaries and thrombus formation in the capillaries, leading to further impairment of microvascular function [29–31]. Increased levels of vasoactive peptides, e.g., endothelin-1, in patients with diabetes might further impair microvascular reactivity in these patients. The production and plasma levels of endothelin-1 are elevated in patients with diabetes, and a positive correlation between plasma endothelin-1 levels and diabetic microangiopathy has been described [32].

Patients with ACS had lower ankle blood pressure than patients without ACS indicating presence of systemic atherosclerosis. However, ABI was not different between the groups. An association between peripheral arterial disease and skin microcirculation has been described [33], but in the present study, there was no correlation between ankle blood pressure or ABI and peak LDF.

We have previously shown a relation between insulin sensitivity and skin microvascular reactivity [34, 35]. Insulin sensitivity is usually related to body mass index (BMI), and the highest BMI in this study was found in patients with ACS and diabetes, which might have influenced the microcirculation in these patients. A non significant negative correlation between BMI and peak LDF was found in this study. We have recently shown that statin treatment improves microvascular reactivity in patients with coronary artery disease and dysglycaemia [34]. The microvascular dysfunction in patients with ACS and diabetes might be even more profound than we have demonstrated in this study since more patients with ACS were on statins.

We studied microvascular function by LDF, a measure of total skin microcirculation, i.e., nutritional capillary blood flow and non-nutritional sub papillary blood flow. An impaired skin maximum hyperaemic response to local heating has been observed in patients with diabetes, and it is associated with macro- and microvascular complications and increased cardiovascular risk [1, 36]. The maximum microvascular hyperaemic response to local heating and arterial occlusion is likely to involve neurogenic and endothelium-dependent mechanisms induced by structural changes in the vessel wall [37].

There are limitations to this study. The study is small, and in particular the number of patients with no ACS was low in comparison with the other groups. Thus, care should be taken when the results are assessed. Furthermore, there was no group with stable coronary artery disease and diabetes to reveal the importance of an ACS for microvascular function. A methodological limitation is high coefficient of variability for LDF as compared to more recent techniques to study skin microcirculation such as laser speckle contrast imaging [38*–*40]*.*

In conclusion, skin microvascular reactivity is impaired in patients with ACS, and the impairment is more pronounced in patients with concomitant diabetes. Diabetes alone has a major impact on microvascular function. A disturbed microvascular function may contribute to impaired myocardial perfusion during ischaemic events and/or PCI. None-invasive methods such as LDF to assess microvascular reactivity are promising tools when new treatment strategies to improve vascular function in patients with coronary artery disease are investigated.
